# Circulars, Bulletins and Reports Issued from the Office of the Chief Surgeon of the American Expeditionary Forces in France

**Published:** 1918-12

**Authors:** 


### CIRCULARS, BULLETINS AND REPORTS

### Issued from the Office of the Chief Surgeon of the American Expeditionary Forces in France.

*Under this heading will be published extracts from circulars and bulletins issued by the Chief Surgeon of the Medical Department of the American Expeditionary Forces in France. It is believed that these will be of general interest and value to medical officers.*

### EXTRACTS FROM C. S. O. CIRCULARS

### Transfusion Sets.

On several occasions requisitions for transfusion sets have been received from Base Hospitals, with the explanation that the transfusion set formerly on hand had been taken to an advanced Field, Evacuation or Mobile Hospital by some member of the staff on detached service with a " shock team ".

The impression has been gathered, apparently, that transfusion sets issued to individuals, upon completing the course in resuscitation at the Central Medical Department Laboratory, were for their personal use. This impression is erroneous, as each set was destined for use in the hospital to which the individual returned, and should have been turned over for use to the Supply Officer of the hospital.

All tranfusion sets now in the possession of individuals will be turned in to the Supply Officer of the hospital to which they are permanently attached. Tranfusion sets have been issued to advanced hospitals, and reserve supplies have been placed in Army Medical Dumps. These supplies are adequate for the use of " shock teams " serving temporarily at advanced hospitals.

### " Shock Teams. "

It is directed that Emergency Medical Teams (Shock Teams), when once formed, be left intact by Commanding Officers of Medical Department units unless specific authority to change personnel of these teams is obtained from the Office of the Chief Surgeon, or from the Director of Professional Services.

### Records to Accompany Patients on Evacuation from Hospitals.

1. Attention of all medical officers is called to the instruction on the field cards, which state that those cards are to be securely fastened to the patient's clothing. Those instructions are not being carried out, and as a result patients and their cards are becoming separated, and there is great confusion of records. In some cases, when patients are being evacuated by hospital trains, the field cards are turned over in bulk to the train commanders. This method of transfer of field cards is not authorized, and train commanders are hereby instructed not to accept field cards in this manner.

2. Many patients are being received at hospitals in Base Ports for evacuation to the United States without adequate records of previous condition. Attention is called to the requirements of G. O. 41, G. H. Q., 1918, Section 1, Paragraph 8; and to the Manual of Sick and Wounded Reports, Sections 6 and 7, and Section 9, Paragraph 12.

3. In making report, disability boards will use card Form 25, Statistical Section, A. G. O.

*Personal Property of Patients.* It has been reported that articles of value have been turned in, without receipt, by great numbers of wounded soldiers at field, evacuation and other hospitals, and that, on their being evacuated to other hospitals, these articles have not been returned to them. Commanding Officers of hospitals should give this matter their attention, and endeavor to see that personal property belonging to their patients accompanies them upon evacuation.

*Fire Protection.* The following suggestion is made to this office by the Bureau of Fire Protection :

"In hospitals where different types of construction have been used, commanding officers should keep in mind, in making assignments of patients to wards, that on account of the difficulties of evacuation in case of fire, the more serious bed patients should, whenever practicable, be placed in less inflammable wards."

*Emergency Medical Teams.* The medical teams heretofore known as "Gas Teams" or "Shock Teams" will be known in the future as "Emergency Medical Teams". They are to be used in emergencies for the medical care of the wounded (especially chest wounds), and for those suffering from surgical shock as well as gas.

*Epidemic Disease.* The attention of surgeons of all organizations and commanding officers of all medical department units is again called to the necessity for prompt report to the local French Civil and Military authorities of all cases of epidemic disease. This report should give the name and organization of patient.

*Clinical Records.* It is desired that the clinical records of patients treated in S. O. S. hospitals be as complete as circumstances will permit. Form 55, Medical Department, will be used for this purpose. Form 55 will be made out for all patients, but only such other parts of Form 55 will be used as are of interest or value in the individual case. The clinical record for complete cases will be filed in the hospital in which the case is completed. When patients are transferred from one S. O. S. hospital to another, Form 55 will be placed in the envelope with the Field Medical Card.

*Rations for Evacuated Patients.* Many cases have occurred recently where patients were evacuated from one hospital to another without sufficient rations. In travel of this sort there are many and unexpected delays. In addition to the cooked rations issued for the expected length of the journey, a reserve of cooked or travel rations for at least 36 hours over and above an ordinary schedule time should be issued for each patient. The number of such travel rations issued can be noted on the travel order and patient is required to turn in rations unused on arrival.

*Patients to be Examined by Board Officers.* It is desired that in the future no patients be transferred from hospital, either to duty or convalescent camp, without having been examined by a board of medical officers. In most cases disability boards already appointed can act upon all such cases. Where the time of disability boards is fully occupied with Class D cases, a Board, to consist of the Chief of Service and Ward Surgeon, can act upon cases going to duty or convalescent camp. Complete physical examination will not usually be required in such cases, and no formal records of the proceedings of the Board other than a note by the senior member on the patient's clinical record.

*Prolonged Active Hospital Treatment.* Patients have recently been evacuated from the Front to S. O. S. hospitals " for continuation of antisyphilitic treatment ". General orders and circulars issued on this subject provide that " only cases presenting complications indicating the necessity of prolonged active hospital treatment will be transferred back from the regimental lines ". In this connection attention of all medical officers is called to Paragraph 5, Section 1, C. O. 34, G. H. Q., 1917, and Paragraph 5, Circular 15, O. C. S., 1917.

*Evacuating Officers and Soldiers from Hospitals.* There have been frequent complaints that orders governing the evacuation of officers and soldiers from hospitals were not being complied with. Commanding officers of hospital centers and hospitals are charged with the duty of seeing that all the officers of their command concerned with the evacuation of patients from hospitals are thoroughly familiar with the orders governing this subject. In this connection attention is called to Section 7, G. O. 111, G. H. Q., 1918; Section 2, G. O. 11, S. O. S., 1918; Section 1, G. O. 41, G. H. Q., 1918; and Circular Letter 6-A O. C.S., 1918.

### Official Relations between Medical and Veterinary Personnel.

The Veterinary Service of the A. E. F. is by S. O. now placed under the authority of the Chief Surgeon, and the Veterinary Corps will, in the future, function under S. R. 70, dated Washington, December 15, 1917.

This Special Regulation is not to be interpreted as placing individual veterinary officers or veterinary organizations under the authority of medical officers. On the other hand, it is to be interpreted as placing all detachments of veterinary personnel in an independent status with reference to other medical department personnel.

The senior veterinary officer of any organization or station, therefore, would bear the same relationship to the commanding officer thereof as does the senior medical officer, and, as a detachment commander, he has the same responsibility for the care, instruction and discipline of his men.

Senior veterinary officers are not to be considered as assistants or subordinates to corresponding medical officers. It is not contemplated that correspondence, reports, or returns emanating from or pertaining to the Veterinary Corps will pass through the office of medical officers as part of the routine channel of communication.

Requisitions for veterinary supplies will be forwarded as follows : -

(*a*) Organizations with divisions through Division Veterinarian, and upon his approval, in the manner as laid down by G. O. 44.

(*b*) O. C. veterinary hospitals and other independent units direct to proper supply depot.

Although the independence of action outlined herein is expected to govern official relations between the Medical and Veterinary Service, it should not be forgotten that the activities of both are in contact at several points, and that frequently occasion arises when the medical officers, by reason of longer service and broader experience, can be of material assistance to the veterinary officer. This is particularly true as regards Army Corps and Division Surgeons and Veterinarians.

Senior medical officers will, therefore, co-operate with veterinarians and assist them by counsel and advice in the handling of duties newer to many of them. While the veterinarian should welcome such assistance, he should at the same time cultivate independence and authority in his department, and avoid submitting himself to such supervisory action as would tend to destroy his initiative and sense of responsibility.

### TELEGRAPHIC REPORTS.

Commanding officers of hospitals, in making telegraphic reports to the British authorities of deaths of British officers and soldiers, should indicate in the report the number or name of the hospital from which the report is being made.

### INSPECTION.

It has been brought to the attention of this office that the isolated detachments connected with divisions and with the S. O. S. sometimes fail to undertake the regular inspections for venereal disease. The attention of all responsible medical officers is called to this oversight.

### TREATMENT OF Y. M. C. A. PERSONNEL.

The requirements of Circular 37, paragraph 8, calling for reports to be submitted to Y. M. C. A. Headquarters for Y. M. C. A. personnel treated in A. E. F. medical formations, are not being observed. In many cases diagnoses are not given, or anything indicating the condition of the patient on discharge from hospital. These reports should be addressed to Medical Section, Y. M. C. A. Headquarters, 12 rue d'Aguesseau, Paris, which change of address will be noted.

### RESUMÉ OF THE SYMPTOMS AND TREATMENT OF POISONING BY IRRITANT GASES.

The gases which have been met with most commonly up to the present time may be divided schematically into three classes :

(1) Suffocative gases, which exercise their main effect on the lung tissue (Chlorine, Phosgene, Diphosgene, Chloropicrin).

(2) Vesicants, the prime effect of which is exercised upon the skin conjunctivitae and upper air passages (Dichlorethyl-sulphide, Mustard Gas or Yperite).

(3) Pure lachrimatory gases (Xylyl-bromide).

Gas may be liberated from cylinders in clouds, a method not now commonly employed, or from shells.

The general aim of the enemy in the present use of gas shells is to fire simultaneously shells of different types, some of which will cause so much sensory irritation that the man will discard his respirator and then become vulnerable to lethal shells, phosgene and similar substances. Owing to this mixture of shells, the symptoms reported by patients are often very confusing."

For this purpose several arsenical compounds have been tried.

*Suffocative Gases.* Suffocative gases, which are relatively non-irritative on inhalation in the concentrations ordinarily used, induce some hours after their entrance an intense œdema of the lungs. Through the great outpouring of fluid into the lung tissue the patient drowns in his own serum; the blood becomes greatly condensed and viscous; there is marked polycythæmia; the capillary flow is obstructed; thromboses are not uncommon; a greatly increased strain is put upon the right heart; the patient suffers from intense oxygen want.

*Sequence of Events.* The immediate effects of irritation of the eyes may be prominent at first, but as a rule quickly pass off; within 3 to 12 hours after exposure to the gas the main symptoms, asphyxia and prostration, due to affection of the lung alveoli and accumulation of fluid in them, appear. In this state the patient's respiration is rapid and usually accompanied by pain (often intense) in the chest; there may be fits of coughing, but the amount of expectoration is very variable, being profuse in some cases, and very scanty is others; in the more severe cases the patient is restless and anxious, or may be semi-comatose with muttering delirium. Therefore many patients will be unable to give a definite account of their symptoms, as loss of memory of immediate events may last for several days. Patients with severe pulmonary œdema fall into two groups:

(*a*) Those with definite venous engorgement. In these the face is congested, the lips blue, and the superficial veins of the face may be visibly distended. There is true hyperpnœa, i.e., the breathing is not only increased in frequency, but the actual amount of air reaching the lungs is greater than normal. The pulse is full and of good tension, and the rate is not often much above 100.

(*b*) Those with collapse. In these the face is pale and the lips of a leaden color. The breathing is shallow, so that there is but little true hyperpnœa. The pulse is rapid (130 to 140) and weak.

In patients who recover, the œdema fluid is absorbed within a few days; in some cases signs of bronchitis or broncho-pneumonia, due to a secondary infection, persist for some time, but in most cases the lung returns to a condition which is normal except for the presence of some disruptive emphysema. In consequence, however, of the œdema of the lungs during the early stage, deficient oxygenation of the blood occurs, unless prevented by the administration of oxygen. The deficient oxygenation gives rise to wide-spread temporary injury in the various systems....

*Vesicants.* The only one hitherto employed is dichlorethyl-sulphide, an oily liquid used in shells, and scattered from them on the ground, where it slowly evaporates. This not only attacks those in the immediate vicinity of the shellburst, but may affect those who may walk over the contaminated ground later. The fluid may be spattered also on clothing, shell-casings, rifles, etc., and may thus become effective through direct contamination of the skin.

The main action of this group is an irritant one on the skin, eyes and respiratory passages.

*Special Symptoms.* (*a*) Early. These are insignificant, nothing being noticed immediately except a smell reminiscent of mustard, from which the gas derives its name (Mustard Gas). A soldier may not realize for many hours that he has been exposed to gas, until the more important delayed symptoms develop.

(*b*) Delayed. These are the principal symptoms of this group and appear 3 to 24 hours after being gassed. They occur usually in the following order, and approximately after intervals stated.

(1) Conjunctivitis. (3 hours.) This rapidly becomes very acute, and is accompanied by intense photophobia, and swelling of the lids, which may cause closure of the eyes for days.

(2) Vomiting and epigastric pain. (4 to 8 hours.) These symptoms appear together as a rule, and are apt to be persistent and intractable.

(3) Burns. (12 hours.) Wide-spread erythema with local vesication occurs going on to definite burns. The commonest sites are the axillæ, genitals and back, but no area may be exempt. The affected surfaces frequently develop very marked pigmentation. Deep burns sometimes occur when the liquid itself comes into contact with the clothes or skin.

(4) Laryngitis, pharyngitis, tracheitis and bronchitis. (24 to 48 hours.) These are the most dangerous symptoms. The degree and extent of the lesion may vary from a simple irritation of the surface to an ulceration of the mucous membrane of the whole passages, followed by infection of the raw surfaces. These conditions may be so extensive and severe as to cause death by themselves or in consequence of the development of broncho-pneumonia.

In a certain number of cases with severe involvement of the respiratory organs, which recover, there has evidently been some interference with the proper oxygenation of the blood, which may give rise eventually to symptoms resembling the after effects of the suffocative gases....

When a soldier is protected by the respirator, the respiratory and eye symptoms are absent or slight.

*Treatment.*

*Suffocative Gases.* The grave symptoms here are due mainly to the intense pulmonary œdema. The conditions which we have to combat are essentially :

(*a*) Oxygen want.

(*b*) Condensation of blood.

(*c*) Overburdening of the right heart.

Our main aims are :

(*a*) Rest.

(*b*) Warmth.

(*c*) Oxygen.

(*d*) Bleeding.

(a) *Rest.* Protect the patient from all unnecessary physical effort in order to reduce the oxygen need. Do not disturb him at the advanced aid station by questioning; his life may depend on the care with which he is handled in the early stage.

*All the gassed should be stretcher cases.* Small oxygen tubes, if available, should be carried in each ambulance in the proportion of one to each stretcher case, and exchanged at the evacuation hospital for freshly filled tubes; these can of course be used only when the ambulance has passed out of the gassed area.

Give the patient fresh air. Do not close the ambulance too tightly, unless it be very dusty.

(b) *Warmth.* Warmth is important. Cold and shivering mean an increased production of $CO_2$ and an increased demand for oxygen. The clothes must be removed at the earliest moment, for they hold gas and may be dangerous not only to the patient but to those about him; warm covering must, however, be provided.

(c) *Oxygen.* The administration of oxygen in all dyspnœic, cyanotic patients is of vital importance. The administration should be as nearly continuous as possible up to the point of the disappearance of the cyanosis, and should be continually repeated whenever the demand is evident.

(d) *Bleeding.* In patients who are cyanotic and show engorgement of the venous system, bleeding is indicated. By venesection we combat :

(1) Oedema of the lungs.

(2) The condensation of the blood ; for with the abstraction of the polycythemic blood, fluid is drawn from the lungs and the tissues, and the circulatory medium becomes less viscous.

(3) The overburdening of the right heart.

The bleeding should be early and free, from 2—600 cc.

Bleeding is inadvisable, even dangerous, in the patient who is pale and in collapse.

If the heart's action be rapid or feeble, bleeding may be preceded by an intramuscular injection, 15 minutes before the venesection of 1/4 milligramme (gr. 1/250) Digitaline cristallisée Nativelle. This may, if necessary, be repeated once or twice in the next 24 hours, and continued later by the mouth if necessary.

In the early stages, during the period of distressing restlessness and agitation and pulmonary edema, morphia may be necessary. Its action as a respiratory depressant is believed by some to be dangerous; and the administration of oxygen, if it suffices, is the safest and best means of quieting the agitation. Where the distress and physical effort associated with the struggles of the patient are great, morphia 0.016 (gr. 1/4), hypodermically, may be demanded, but at the same time it should be remembered that in collapse, dulling of the respiratory centre may turn the scale against the patient.

*Treatment of the Pale, Grey Cases with Collapse.* Oxygen is here the main aim, and the administration should be practically continuous.

Never bleed these patients. Bleed only those with venous congestion.

Rest, warmth and oxygen are the main stays of treatment. Atropine and adrenaline are contra-indicated. These drugs place an increased strain on the heart. It is best to abstain from intravenous salt solution injections. The fluid introduced puts an extra burden on the heart, is soon absorbed into the tissues, and may increase the pulmonary edema. In grave cardiac weakness, preparations of camphor or caffeine may be given hypodermically and digitalis may be indicated, according to the nature of the case.

*Relapses.* In any patient who has had pulmonary edema it may, within the first few days, recur on slight exertion or even without apparent cause, and if there have been any definite symptoms of edema of the lungs, the patient should be kept in bed for a week.

Smoking should be absolutely prohibited, and convalescents should not be allowed to smoke in the ward in which these patients lie.

Patients whose symptoms have been mild should, if possible, be put on graduated exercises as soon as they are out of bed, and under military discipline as soon as possible. Mild cases should be back in the line in about two weeks. Severe cases may have to remain in the hospital for three or four weeks, and thereafter spend several weeks in a convalescent camp.

Great care should be taken to protect the convalescent from secondary infections. Wherever it is possible, beds should be isolated one from another by sheets, as in acute respiratory infections, for secondary bronchitis and broncho-pneumonia are not uncommon and the danger of cross infection should be provided against.

*Vesicant Gases.* The symptoms, here, are usually delayed from three to twenty-four hours, and dangerous symptoms do not as a rule appear for from twenty-four to forty-eight hours after exposure, but pulmonary edema and symptoms similar to those observed in the suffocative cases may occur; moreover, the patient may have had a double exposure to different sorts of gas. Therefore, all the precautions above mentioned should be observed at the outset, but other special steps must be taken.

*Disposition of Clothes.* Wherever exposure to a vesicant gas is suspected, the use of external warmth should be avoided if the clothes have not previously been removed. The application of heat favors the diffusion of the gas.

Remove the clothes as soon as possible, but protect the patient from exposure during the process.

After removal the clothes should be sterilized in wet steam for 30 minutes; in dry heat for 15 minutes; and exposed to the air for 15 minutes. This may be carried out in the Thresh sterilizer, and may have to be repeated twice, although two or even one treatment may be efficacious. While waiting for sterilization, have the clothes placed outside the quarters, in the open. All who handle the clothes must be protected by respirators, special oiled clothing and gloves.

*Removal of the Poison from the Skin.* The patient should be thoroughly bathed in soap and water in a warm room at the earliest possible moment. Areas which have been specially exposed may first be covered for a few minutes by a paste of 25-50 o/o chloride of lime in water, and then washed with warm water. Bathing with 0.05 o/o permanganate of potassium is said to be useful.

*Treatment of the Skin and Mucous Membranes.* When the skin is dry, erythematous areas may be powdered with subnitrate or subcarbonate of bismuth — oxide of zinc — talcum, or any simple non-irritating powder. Moist and raw surfaces may also be powdered with the same substances, or a powder consisting of :

| | | Grammes |
|---|---|---|
| Oxide of Zinc | } | 200 |
| Carbonate of Magnesia | } | |
| Carbonate of Lime | } | |
| Talcum Powder | | 400 |

and protected from the bed clothes by cribs, or covered by a non-absorbent dressing.

If a moist dressing be preferred, a solution consisting of

| | Grammes |
|---|---|
| Sodium Chloride | 70 |
| Sodium Bicarbonate | 150 |
| Water | 5000 |

may be used — simply lime water.

Blisters should receive careful attention. The contents of the vesicles are poisonous and irritating to the surrounding skin; the blisters should, therefore, be opened carefully and the contents taken up with absorbent cotton, which should promptly be burned. Interdigital areas should be washed carefully each day, powdered and bandaged.

Fatty salves, in the early stages, are inadvisable, as any undestroyed poison which remains on the skin may be diffused underneath.

Later, deep and painful burns are much relieved by treatment with ambrine.

The eyes should be irrigated immediately with warm alkaline solutions, such as the above mentioned solution of sodium chloride, sodium bicarbonate and water. After this, some non-irritating oil, such as liquid *albolene*, should be instilled. The patient should be kept in a dark room, or the eyes shaded. Compresses soaked in this solution may give comfort in the acute stage. In severe cases, frequent (every 2-3 hours) irrigation of the conjunctiva with simple boric solutions (Sodii Boratis 0.65) (Aquæ Camphoræ 30), followed by the instillation of liquid albolene, should be carried out.

The nose should be sprayed with a warm alkaline solution (sod. chloride, sod. bicarbonate and water, as above) and also with liquid albolene, to which a little menthol may be added (such as the preparation known as " Chloretone Inhalant ").

The mouth should be rinsed with alkaline washes and gargles.

The laryngeal inflammations may be relieved by inhalation of:

| | |
|---|---|
| Menthol | 0.65 |
| Tinct. Benzoini Comp. ad | 30 |

of which 5 cc. are added to 500 cc. steaming water.

*Secondary Respiratory Infections and Sequels of Gas Poisoning.* "Mustard" cases may develop grave secondary bronchitis with broncho-pneumonia. In the treatment of such instances there is nothing specific. Every precaution should, however, be taken to prevent cross-infection. The beds of all patients with purulent bronchitis and broncho-pneumonia should be screened one from another, and from their neighbours.

In soldiers who have been "gassed", especially with phosgene, symptoms similar to those characterizing D.A.H. (Effort Syndrome) are not uncommon,—dyspnœa on exertion, pain in the chest, palpitation, dizziness, fatigue on exertion, disturbed sleep with dreams, paroxysms of coughing and even asthma-like attacks. These patients are often polycythemic. Nervous manifestations unassociated with apparent organic lesion are common.

Get these patients out of bed and start carefully graduated exercises, sending them as soon as possible to a special training camp.

"Functional" photophobia and blepharospasm are frequent, but eye shades and colored glasses should be discontinued as soon as the acute inflammatory stage is over. When this has passed, the use of eye drops of a solution of:

| | | |
|---|---|---|
| Zinci Sulphatis. . . . . . . . . . | 0.065—0.13 | (gr. I-II) |
| Acidi borici. . . . . . . . . . . . | 3.75 | ( ʒ I ) |
| Aquæ. . . . . . . . . . . . . . . | 30 | ( ʒ I ) |

is said to give relief. If corneal ulcers or iritis, which are not common, be present, they must be treated in the usual manner. Threatening though the ocular manifestations may be, recovery is usually complete. Grave damage to the uveal tract is rare. It is important not to overtreat the eyes.

In all cases preserve an optimistic attitude; the great majority of gassed patients recover completely.

Do not let the patients become introspective or "hospitalized". Keep them occupied in mind and body. Get the "mustard" gas cases who have no respiratory involvement out of bed in two or three days if possible. Remove the eye shades as soon as the acute inflammatory stage is over. Send the men out of doors, look out for their employment or amusement, and get them under army discipline as soon as may be. Far too many convalescent "gassed" cases tend to accumulate, uncared for, in base hospitals. The responsibility of the medical officer does not end with the disappearance of the dangerous symptoms. See to it that the patient does not become a psychoneurotic.

Attention to these details may save a considerable wastage of men.

### EXTRACTS FROM C. S. O. BULLETINS

*The Care of Wounded Men. 35th Division Memorandum to Regimental Battalion Surgeons.* Extracts of particular importance. There are three main factors to be considered in the care of wounded men, namely, — hemorrhage, pain and cold. Hemorrhage should be controlled first, and at the time the wounded are first seen. They should then be transported to the nearest aid station where all fractures should be properly splinted, the wounds dressed, and the patients warmed and given hot drinks. The administration of warmth is of the greatest importance in the prevention of shock. This will be secured by the use of blankets, stoves, hot water bags, hot bottles, etc. Patients will not be removed from the litter, but new litters, with a number of blankets and other appliances sent down from the front line trenches, will be returned by the bearers to replace those used.

An effort should be made to control hemorrhage without using the tourniquet. If there is doubt as to whether or not the bleeding has been controlled, a tourniquet may be loosely applied and left in position. If absolutely necessary, it may, of course, be left on tight. In either case the fact should be noted on the diagnosis tag and the litter bearers informed of the condition.

Never forget that, in the final analysis, the prime and ultimate object of the Medical Department is to assist in the conservation and maintenance at the highest possible point of the fighting efficiency of the line troops. If the men become sick, get them well by the best means possible and in the shortest possible time; if wounded speed their evacuation to hospitals, where definite treatment may be given. Prepare them in every way, by every means available in the front line stations, for evacuation; get them back in the best possible condition. Hold malingerers to their work, but exercise judgment with the slightly ailing men; frequently two or three days' rest treatment in the field hospital will save to the service men with slight indisposition who, if held in the trenches, might go on to a severe illness, or to a total loss. The condition in which men are received in the field hospitals from the front lines is always indicative of the efficiency of the men in the advance sectors. It has been noted that some of the diagnosis tags are not being properly made out, and that even in the back areas they are often signed by enlisted personnel of the sanitary units. This is, of course, permissible in emergencies, but whenever possible diagnosis tags should be carefully looked over and signed by the medical officer.

*Gas Fright.* Gas fright does not require evacuation. In a recent attack 261 patients were sent to the field hospital as gassed cases. Over half of these were not sufficiently gassed to require their evacuation from the front line. There were several who manifested no evidences of gas poisoning when received at the field hospital.

It is necessary that surgeons use great discretion in evacuation of gassed cases, in order to give prompt and effective treatment to those who are sufficiently gassed to be incapacitated, and to prevent depletion of effectives by evacuation of persons who are perfectly able to carry on. Where concentrated gases are employed, short exposure only is required to produce severe poisoning. Where gases are highly diluted, it often happens that the men may be exposed for some time and still be able to continue on duty.

Many men are suffering not from gassing but from gas fright All they need is reassurance. Surgeons must base their diagnosis upon physical signs rather than upon statements made by prospective patients. Place more reliance upon sounds made by the patient's lungs than upon sounds made by his mouth. Have at the regimental aid stations a place for slightly gassed similar to the stations for slightly wounded. Here cases about which there is question may be rested and observed for a short time. Many of them will be able to return to duty. Malingerers will, of course, be immediately returned to the front.

When working in gas infected areas for a long time, all troops may be more or less gassed. It is for the surgeon to estimate the degree of gas poisoning in each case, taking into consideration the kinds of gases used, their concentration, amount of exposure, physical signs manifested by the patient and the exigency of the military situation.

*Advice on Field Medical Service from. ... Division.* Incident to the attack and capture of the village of ... the following observations were made :

(*a*) A potential psychic advantage was demonstrable in instances where casualties occurred, when medical department personnel were in close proximity or easily available.

(*b*) The necessity for observing body and clothing cleanliness for the purpose of reducing infection complications as a result of wounds.

(*c*) The necessity for increasing first-aid and litter-bearing personnel.

(*d*) The lack of adaptability of litters other than the horse-shoe or bamboo sling type for trench evacuation of wounded.

(*e*) The advantages gained by reaching wounded promptly, and thereby evacuating them more rapidly.

(*f*) The necessity for having sturdy, strong personnel for litter-bearing work.

(*g*) Close liaison between Battalion Aid Stations and ambulance centers.

(*h*) The lack of propriety in the application of tourniquets, unless it is certain that their removal may be accomplished within one hour at a maximum.

(*i*) The importance of commissioned and first-aid medical personnel accompanying attack waves.

(*j*) A careful and intelligent discrimination between gas poison and gas fright, and shell shock and shell fright, with the appropriate disposal of each classification.

(*k*) The establishment of a place adjacent to Battalion or Regimental Aid Station, depending upon battle conditions, where rest and nourishment may be accomplished, followed by return to the line without the loss of time, instead of evacuation to the rear.

It was observed that many men were evacuated to a Field Hospital who were suffering from fatigue and hunger. By applying nourishment and permitting rest, they would have been available for front line work within three hours, and the conservation of this man power is clearly desirable.

*A Reminder for Surgeons Suggested by the State of Mind and Body of Some Recently Operated Cases.* Surgeons must free themselves from their tendency to treat the wounds and forget the function; to make a well man but not a working man; to take the anatomical rather than the physiological point of view.

In amputated cases, weight-bearing must begin at the earliest possible moment that the condition of the stump will allow. It is better for the physical condition of the stump and the mental condition of the soldier, and prevents the muscular contraction which makes later fitting of apparatus impossible until it is overcome. Experience has shown that from one-third to one-half of the ordinary deformities resulting from gun-shot wounds are preventable. Though the ability to exercise preventive measures is never a question of minutes, it is often a question of a few days. There is a psychological moment in convalescence when the soft callus of a bowing bone is mouldable to proper alignment, when a contracting joint may be gradually and safely straightened without suffering or operation. The demand for speedy evacuation from the temporary hospitals, where the greater part of the surgery is done, focuses attention on the immediate results. The surgeons upon whom, in the various later stages of convalescence, the care of the case devolves, feel a certain lack of responsibility for the functional result of the first operation, and devote their chief attention to the general condition of the patient and the healing of his wound, since the pressure for beds in these general hospitals is only slightly less than in the front lines.

### Cellulose Tissue for the dressing of Wounds — A Substitute for Gutta-Percha tissue.

The custom is practically universal in the Forward Hospitals in the A. E. F. to dress wounds which have been subjected to primary debridement with dry sterile gauze, and to utilize dry sterile gauze for drains and wicks. When Carrel-Dakin solution is available, these dressings may be kept moist, but in the larger number of cases it is not available, and the secondary dressings with removal of the gauze are extremely painful. The gauze, moreover, does not drain wounds as it is supposed to do, and its extraction after having been packed into a wound dislocates freshly severed tissues, leads to bleeding, and re-opens possible avenues of infection.

In many clinics at home the placement of gauze against a raw wound is prohibited, some tissue like gutta percha tissue always being inserted between the gauze and the raw surface, or if a wick or drain is to be used the gauze is rolled in this tissue, cigarette fashion. Gutta-percha tissue has been used in France in certain hospitals which brought a supply with them, but the available amount of this material is scanty. A substitute for gutta-percha tissue during the past years has been used by many French surgeons in the form of " cellophane ", and an equally good substitute is obtainable from the Medical Supply Depots, A. E. F., in the form of " Cellulose " tissue. It is stated that in requisitioning for this tissue it should be designated as " Cellulose Tissue ", substitute for oiled silk.

This tissue stands up well under contact with all antiseptics in wet dressings and irrigations, except that after 24 hour contact with the chlorine preparations, such as Dakin solution and chlorozone, it loses some of its texture and should be changed. It is strengthened rather than weakened by sterilization by heat.

Medical officers who are interested in improving the condition of the wounds which come under their care, and in saving the pain of secondary dressings which often necessitate an anesthetic, will be glad to know that a satisfactory protective substance is thus available.

*Sanitary Score Card.* The following score card is posted in a conspicuous place in each kitchen. The scoring is made by the Sanitary Inspector, or one of his assistants. Each heading is marked on a scale of (10) ten. Score cards are collected weekly, copies are furnished organization commanders, district commanders, and the Division Surgeon. A consolidated weekly report is published in orders by the Division Commander. This statement enables all concerned to tell at a glance the sanitary conditions of individual units. Both points of excellence and delinquencies are recognized, so that a spirit of rivalry exists which extends from cooks and kitchen police to organization commanders. Since the inauguration of this system, greatly improved sanitary conditions of organizations have been noted.

*Organization.*

| | Mon. | Tues. | Wed. | Thurs. | Fri. | Sat. | Sun. |
|---|---|---|---|---|---|---|---|
| 1. Cleanliness of kitchen and kitchen utensils . . . . . . . . . . . . | | | | | | | |
| 2. Care of food stuffs . . . . . . . . . | | | | | | | |
| 3. Menu and conservation of food . . . | | | | | | | |
| 4. Cleanliness of kitchen personnel . . | | | | | | | |
| 5. Care of garbage cans. . . . . . . . | | | | | | | |
| 6. Disposal of liquid wastes. . . . . | | | | | | | |
| 7. Condition of water for drinking . . | | | | | | | |
| 8. Sanitation of billets . . . . . . . . | | | | | | | |
| 9. Sanitation of latrines. . . . . . . . | | | | | | | |
| 10. Bath record . . . . . . . . . . . . | | | | | | | |

*In Bags (Lyster) Not Bottles is Your Safe Drink in France.* Various brands of bottled table waters, some of which claim medicinal properties, and all of which purport to be pure, contain sufficient numbers of intestinal bacteria to be unsafe for use. Furthermore, it is learned that soft drinks are not uncommonly prepared with water which is not marked "water for drinking", or chlorinated and therefore unsuitable for use by our soldiers, and forbidden to them. The ingredients, syrups, flavors, etc., added to the waters in the making of soft drinks, do not suffice to make polluted or unsafe water fit to drink. The A. R. C., K. C., Y. M. C. A., and Y. W. C. A. have been officially warned that before offering any waters or drinks, bottled or otherwise, as free distribution or for sale to members of the A. E. F., approval of the water supply must be obtained from the medical officer responsible for the sanitation of the area within which the distributing station is operating. The medical officer will, where necessary, obtain the necessary laboratory examination of water from bottle, spring, well or other supplies.

A drink recently offered as "Bill's Bug Juice" contains too many toxic possibilities to be wholly hygienic, even though the raspberry constituent was from real fruit.

*Salvaging and Washing Gauze, as Practiced at a Base Hospital.* The following method of salvaging and washing gauze, modified from methods in use in civil hospitals in the States, will save 65 to 70 o/o of used gauze and bandages for re-use. By this method dressings and bandages can be rewashed many times. The process can be handled entirely by convalescent patients, with the supervision of one or two N. C. O. or enlisted men of the Medical Detachment, who can also run the portable sterilizer : —

(*a*) All dressings removed from wounds are saved in G. I. cans, in surgical wards, in dressing rooms, and in operating rooms. These are collected daily and taken to gauze salvage station, which station should house a portable sterilizer to furnish steam for washing. (b) *Sorting.* On receipt at salvage station, dressings are sorted, and gauze and bandages separated from cotton and other foreign bodies; all bandages and gauze are saved for washing, except gauze heavily loaded with boric ointment or picric acid. (c) *Rinsing.* The gauze and bandages, after sorting, are placed in a large concrete tank of cold water, which is frequently agitated, and changed twice in 24 hours. This is for the purpose of rinsing blood and pus from the dressings. (d) *Washing.* After rinsing for 24 hours, gauze is separated from bandages and washed separately in an ordinary steam washer. If the revolving type is used, the gauze will have to be placed in cord bags, commonly used by steam laundries in washing collars. If the gauze is washed in a small washer of the "Dolly" type, it will not be necessary to place in cord bags. Bandages are similarly handled in washing, but are washed separately from the gauze. Wash each load one hour with live steam from sterilizers. (e) *Second Rinsing—Drying.* On removal from washer, they are carefully rinsed in tepid water and wrung through hand wringers by patients. They are then hung on lines out of doors to dry, or, in wet weather, they are dried in a closed room that is heated by a stove. When nearly dry, they are picked over and spread out in piles of equal size. (f) *Bulk Sterilization.* They are then sterilized in bulk for one hour, sent to the Surgical

Supply Room, sorted into small packages, and re-sterilized in autoclaves for use in operating rooms, dressing rooms, and for ward dressings. In civil hospital practice, in addition to the above, gauze is usually passed through a bleaching solution of calcium chloride and soda. In the present emergency, this step can be omitted without detriment.

*Sanitary Report ... Division*, contrasts the dark, dirty, unsanitary billets, surrounded by manure heaps and infested by fleas and lice, with the well policed tent groups where infection is less easily communicated and living conditions are in all respects superior. His recommendation " that all men in billets be taken out and placed under canvas, except where billets are unusually good, or area for tentage is too limited or exposed ", was endorsed and carried out by Major-General Commanding.

